# Clinical application of the transseptal puncture technique using a biplane positioning method with left atrial 3D-CT

**DOI:** 10.3389/fcvm.2026.1610164

**Published:** 2026-04-14

**Authors:** Yi-Chen Yang, Jun Hou, Yi Li, Tan Chen, Ya-Feng Zhou, Rui-Qing Dong

**Affiliations:** Department of Cardiology, The Fourth Affiliated Hospital of Soochow University, Suzhou, China

**Keywords:** atrial 3D-CT, atrial fibrillation, biplane positioning, cardiac intervention, transseptal puncture

## Abstract

**Introduction:**

Traditional transseptal puncture (TSP) relies on x-ray imaging and anatomical landmarks, which poses challenges in patients with atrial structural variations or overweight. Furthermore, emerging interventional techniques demand precise puncture site localization. This feasibility study evaluates the safety and efficacy of a novel biplane positioning method guided by left atrial (LA) 3D-CT reconstruction for TSP.

**Methods:**

A retrospective analysis included 100 atrial fibrillation patients undergoing radiofrequency catheter ablation (RFCA) between July 2023 and March 2024. Preoperative LA-enhanced CT scans were performed to reconstruct 3D models. Key measurements included vertebral height (H), horizontal distance (X) from the target puncture point (O) to the anterior spine edge at 45° right anterior oblique (RAO) view, and vertical distance (Y) between O and the great cardiac vein. Intraoperative biplane localization integrated CT-derived ratios (X/H, Y/H) with fluoroscopy. Statistical analyses compared outcomes across LA size subgroups.

**Results:**

All patients achieved successful TSP without complications (e.g., cardiac tamponade, thromboembolism). The mean X/H and Y/H ratios were 0.8 ± 0.2 and 0.5 ± 0.1, respectively. Patients with larger LA diameters (≥50 mm) exhibited significantly greater X values (16.8 ± 3.3 mm vs. 13.6 ± 4.2 mm, *P* = 0.034). In 17 patients with unclear LA posterior borders on fluoroscopy (mean BMI 27.2 ± 3.5 vs. 24.9 ± 3.2 in others, *P* = 0.009), the method ensured safe puncture. The mean distance from the puncture site to the right inferior pulmonary vein was 24.2 ± 5.5 mm.

**Discussion:**

The LA 3D-CT-guided biplane positioning method demonstrates feasibility, accuracy, and safety for TSP in atrial fibrillation patients, including those with enlarged atria, structural anomalies, or overweight. The protocol is feasible within a limited, single-center cohort.

## 1 Introduction

Atrial septal puncture is a commonly employed technique in clinical cardiac interventional therapy. Traditional atrial septal puncture primarily relies on x-ray imaging for puncture site localization. Although this technique is relatively mature, it still faces several technical limitations: high dependency on the “fossa ovalis bounce sign,” with some patients exhibiting diminished or absent signs due to atrial structural variations (e.g., atrial septal aneurysm, left atrial enlargement-induced septal bulging) 1, 2, which complicates anatomical localization. Additionally, x-ray visualization of the posterior left atrial border is difficult in obese patients.

Furthermore, emerging interventional technologies such as left atrial appendage closure (LAAC) and transcatheter mitral valve edge-to-edge repair (TEER) require distinct puncture site specifications compared to traditional atrial fibrillation ablation, rendering conventional puncture methods insufficient for modern procedural needs 3–7.

Through two-dimensional imaging of contrast-enhanced left atrial CT and three-dimensional cardiac reconstruction, clinicians can intuitively assess cardiac anatomy to guide atrial septal puncture with precision. This feasibility study aims to evaluate the safety and clinical efficacy of the biplane positioning method through systematic observation and analysis of 100 cases performed at our center.

## 2 Method

### 2.1 Study population

This study enrolled 100 patients who underwent radiofrequency catheter ablation (RFCA) for atrial fibrillation (AF) at the Fourth Affiliated Hospital of Soochow University between July 2023 and March 2024, with preoperative left atrial (LA) enhanced CT scans.

Exclusion criteria: ①Malignant tumors or other severe comorbidities with an expected survival <1 year; ②Intracardiac thrombus detected by preoperative transesophageal echocardiography (TEE); ③Hyperthyroidism; ④Rheumatic heart disease with moderate-to-severe aortic/mitral stenosis or severe mitral regurgitation;⑤Previous AF ablation history, atrial septal closure, or surgical repair;⑥Contraindications to CT enhancement examination(e.g., contrast allergy, renal insufficiency, eGFR < 30 mL/min/1.73m2).

### 2.2 Method of left atrial contrast-enhanced CT imaging

The CT imaging data were acquired on uCT960 scanner (LianYing, CHINA) or Revolution CT scanner (GE, USA) with a single detector width of 160 mm, axial scanning, tube voltage ranging from 80 to 120 kV (adjusted according to the patient's BMI), rotation time of 0.25 s, slice thickness of 0.5 mm, and a matrix of 512 × 512. The patient's elbow vein was injected with a nonionic contrast agent, iohexol (350 mgI/mL), at a rate of 4–5 mL/s using a single-barrel high-pressure injector, with a dose of 0.8 mL/kg. The subject was placed in a supine position and underwent end-inspiratory breath-holding scanning with a breath-holding time of 4–7 s. The scanning range extended from the ascending aorta to the cardiac apex.

### 2.3 Puncture point localization

**Figure 1:**
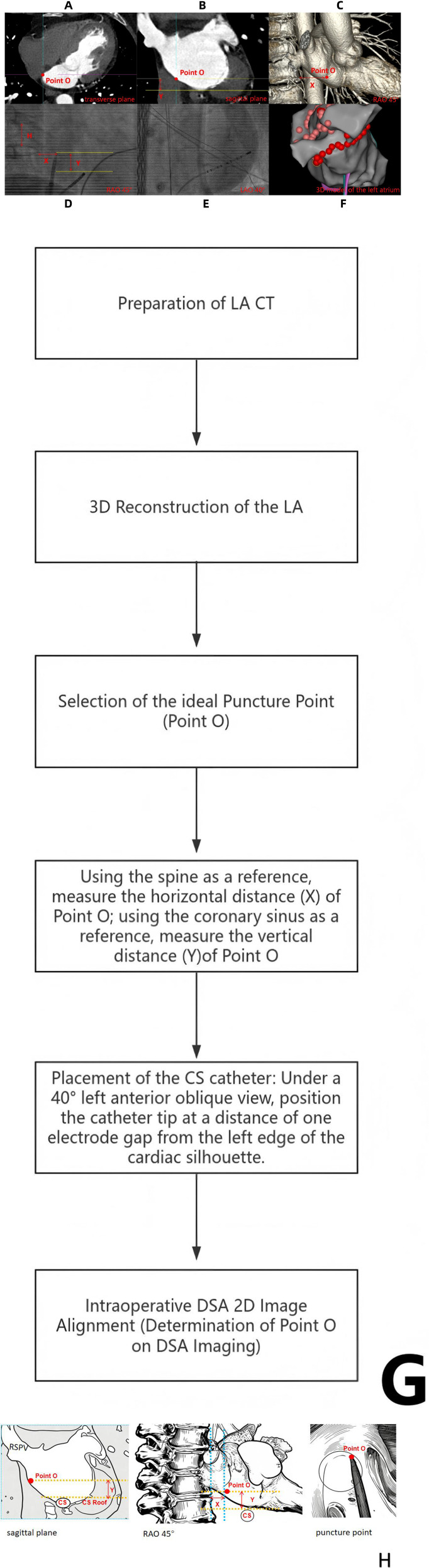
**(A)**: Select the ideal puncture point O in a CT transverse plane; **(B)**: Measure the distance Y (mm) between the highest point of the great cardiac vein and the horizontal line at point O; **(C)**: Measure the distance X (mm) between point O and the anterior edge of the spine at RAO 45°; **(D)**: Placement of the CS catheter; **(E)**: Determination of Point O on DSA Imaging; **(F)**: Use mapping catheter to construct a three-dimensional model of the left atrium through the Carto system; **(G)**: Technical Flowchart; **(H)**: Schematic diagrams).

The atrial septum is a membranous structure located between the left and right atria, consisting of two layers of endocardium, a small amount of myocardial tissue, and connective tissue. The fossa ovalis is the thinnest part of the atrial septum and represents the ideal puncture site for atrial septal puncture 8, 9. When scanning from the superior to the inferior aspect of the left atrium in a CT transverse plane, the point where the atrial septum becomes thinnest corresponds to the fossa ovalis, with this thinnest point defined as the target puncture point (point O) (see Figure 1A). The highest position of the great cardiac vein is identified in the CT sagittal plane, which is the highest position for catheter placement after coronary sinus catheterization during the procedure. The distance Y (mm) between the highest point of the great cardiac vein and the horizontal line passing through point O is measured (see Figure 1B), along with the inner diameter D (mm) of the great cardiac vein in this sagittal plane. Simultaneously, the height H (mm) of a single vertebral body adjacent to point O is measured. Using the three-dimensional reconstruction model of the left atrium, the spatial orientation is adjusted to a right anterior oblique angle of 45° (i.e., the standard fluoroscopic imaging angle used during atrial septal puncture), and the two-dimensional planar distance X (mm) between point O and the anterior edge of the spine is measured at this 45° RAO projection (see Figure 1C).

### 2.4 Working flow

All patients received oral anticoagulant therapy continuously throughout the perioperative period. Following local anesthesia, the right femoral vein was punctured, and a 6F sheath was inserted to deliver the coronary sinus catheter. The catheter tip was positioned approximately 1 cm (equivalent to one catheter spacing) from the left edge of the cardiac silhouette under a 40° left anterior oblique (LAO) fluoroscopic view (see Figure 1D). The Brockenbrough (BRK) needle (Abbott Medical, USA) has been the standard needle used to puncture the patent foramen ovale (PFO) mechanically. The transseptal sheath SL1 (Abbott Medical, USA) was advanced over a guidewire into the superior vena cava under anteroposterior (AP) fluoroscopy, and the transseptal needle was then advanced to the distal end of the sheath without protruding beyond it. The sheath-needle assembly was subsequently retracted into the right atrium with a clockwise rotation. The target O point was determined using the biplanar method: Measuring the horizontal relative distance and vertical relative distance on CT (distance was described in terms of vertebral height, relative distance). The puncture height is adjusted by retracting the long sheath and puncture needle, and the puncture point is adjusted forward and backward by rotating the long sheath and puncture needle clockwise and counterclockwise. The retraction height is determined by the CT measurement value of Y/H; Under a 45° right anterior oblique angle of DSA, referring to the anterior edge of the spine, the puncture point is determined forward and backward based on the CT measurement value of X/H, and the long sheath and puncture needle are adjusted accordingly clockwise or counterclockwise (see Figure 1E). Once the target O point was established, the puncture needle was advanced through the fossa ovalis. Correct entry into the left atrium was confirmed by contrast injection. The long sheath was then slowly advanced into the left atrium, and the needle was withdrawn. A long guidewire was subsequently advanced into the left superior pulmonary vein, followed by further advancement of the long sheath to complete the transseptal puncture. A mapping catheter was introduced via the Carto system to construct a three-dimensional left atrial model (see Figure 1F). The actual distance between the puncture site and the right inferior pulmonary vein was measured, and electrical potentials from the left superior, left inferior, right superior, and right inferior pulmonary veins were mapped to identify the left atrial appendage and pulmonary vein ostia. A radiofrequency ablation catheter was then deployed to perform wide-area circumferential isolation of the left and right pulmonary veins (ablation parameters: power 40–45 W, temperature 47 °C, cold saline flow rate 17 mL/min) until pulmonary vein potentials were completely eliminated. The entire procedural workflow is illustrated in the flowchart (see Figure 1G) and the schematic diagrams (see Figure 1H).

### 2.5 Statistical analysis

Statistical analyses were performed using SPSS 27.0 (IBM Corp.). Continuous variables were assessed for normality using the Shapiro–Wilk test. Normally distributed data are presented as mean ± standard deviation and compared using the independent samples t-test for two-group analyses or the Least Significant Difference (LSD) t-test for multiple independent group comparisons. Non-normally distributed data are reported as median (interquartile range) and analyzed using the Wilcoxon rank-sum test for two-group comparisons or the Mann–Whitney U test for multiple group comparisons. Categorical variables are expressed as frequency (percentage). A two-sided *p*-value < 0.05 was considered statistically significant.

## 3 Results

### 3.1 Baseline information of patients

A total of 100 patients were included, with a mean age of 67.4 ± 9.7 years, mean height of 166.4 ± 8.4 cm, mean weight of 70.1 ± 11.1 kg, and mean BMI of 25.3 ± 3.3. Among them, 68 (68%) were male, and 59 (59%) had persistent atrial fibrillation. Comorbidities included hypertension in 59 patients (59%), diabetes in 5 (5%), coronary heart disease in 16 (16%), prior stroke in 11 (11%), and cardiac insufficiency (LVEF < 50%) in 15 (15%) (see Table 1).

**Table 1:** Baseline data of patients.

project	*n* = 100
Age (years)	67.4 ± 9.7
Height (cm)	166.4 ± 8.4
Weight (kg)	70.1 ± 11.1
BMI（kg/m2）	25.3 ± 3.3
Males (n, %)	68（68%）
Persistent atrial fibrillation (n, %)	59（59%）
Hypertension (n, %)	59（59%）
Diabetes (n, %)	5（5%）
Coronary heart disease (n, %)	16（16%）
Stroke (n, %)	11（11%）
Cardiac insufficiency (n, %)	15（15%）

### 3.2 Transseptal puncture data

Preoperative transthoracic echocardiography in all 100 patients revealed a mean left atrial diameter of 44.5 ± 4.5 mm. Preoperative CT measurements showed a mean vertebral body height (H) adjacent to the ideal puncture point (O point) of 18.0 ± 1.9 mm and a mean internal diameter of the great cardiac vein at its highest point of 11.0 ± 2.5 mm. The mean two-dimensional horizontal distance (X) between the O point and the anterior edge of the spine was 14.0 ± 3.8 mm, and the mean vertical distance (Y) from the O point to the horizontal line through the highest point of the great cardiac vein was 9.7 ± 2.5 mm. The mean X/H ratio was 0.8 ± 0.2, and the mean Y/H ratio was 0.5 ± 0.1. The mean distance from the actual puncture site to the right inferior pulmonary vein was 24.2 ± 5.5 mm (range: 14.4–38.5 mm) (see Table 2). In 17 patients, the posterior border of the left atrium was not clearly visualized under fluoroscopy during transseptal puncture. These patients had a significantly higher mean BMI (27.2 ± 3.5) compared to the remaining 83 patients (24.9 ± 3.2; *P* = 0.009).

**Table 2:** Transseptal puncture data.

project	*n* = 100
Left atrial diameter (mm)	44.5 ± 4.5
Vertebral height H (mm)	18.0 ± 1.9
Inner diameter of great cardiac vein (mm)	11.0 ± 2.5
Distance X (mm) between point O and the anterior edge of the spine	14.0 ± 3.8
Distance Y (mm) between point O and the great cardiac vein	9.7 ± 2.5
X/H	0.8 ± 0.2
Y/H	0.5 ± 0.1
Distance between puncture point and right inferior pulmonary vein (mm)	24.2 ± 5.5

Taking into account the influence of left atrial (LA) diameter on the distance between point O and the anterior spinal edge, patients were stratified into three groups: Group 1 (LA ≤ 40 mm), Group 2 (40 mm < LA < 50 mm), and Group 3 (LA ≥ 50 mm). Group-specific data were analyzed accordingly (see Table 3). Notably, the X value in Group 3 was significantly greater than in Group 1 (*P* = 0.034) and Group 2 (*P* = 0.016).

**Table 3:** Statistical data grouped by left atrial diameter size.

project	Group 1 (*n* = 17)	Group 2 (*n* = 73)	Group 3 (*n* = 10)
Left atrial diameter (mm)	37.7 ± 2.1	44.9 ± 2.4	52.6 ± 1.7
Vertebral height H (mm)	18.3 ± 2.0	17.7 ± 1.8	19.1 ± 2.1
Inner diameter of great cardiac vein (mm)	10.1 ± 2.6	11.0 ± 2.4	12.0 ± 2.7
Horizontal Distance X (mm) between point O and the anterior edge of the spine	13.6 ± 4.2	13.8 ± 3.6	16.8 ± 3.3
Vertical Distance Y (mm) between point O and the great cardiac vein	9.9 ± 2.1	9.5 ± 2.6	10.7 ± 2.4
X/H	0.8 ± 0.2	0.8 ± 0.2	0.9 ± 0.2
Y/H	0.5 ± 0.1	0.5 ± 0.1	0.6 ± 0.2
Ideal Puncture Site Determination Distance between puncture point and right inferior pulmonary vein (mm)	20.5 ± 4.3	24.7 ± 5.3	27.2 ± 5.7

### 3.3 Results of atrial septal puncture

In this retrospective study, transseptal puncture guided by the biplane positioning method using left atrial 3D-CT reconstruction was successfully performed in all 100 patients, with no puncture-related complications such as pericardial effusion, aortic injury, or left atrial posterior wall perforation, thereby confirming the safety and efficacy of this approach.

## 4 Discussion

In 1959, Ross 10 first reported the clinical application of transseptal puncture (TSP). Subsequent refinements to needles, sheaths, and techniques were introduced by Brockenbrough 11 and Mullins 12. Although Ross's classical TSP method remains foundational 13, Chinese investigators have proposed the “left atrium-spine localization method” 14. This method defines the puncture site height as one vertebral body height above the inferior border of the left atrial shadow along the spinal midline under posteroanterior fluoroscopy; under 45° RAO fluoroscopy, the site is located between a position one vertebral body height anterior to the posterior cardiac border and the midpoint connecting the posterior cardiac border to the atrioventricular groove shadow; and the needle/sheath configuration requires straightening of the distal curvature into a linear or near-linear shape during puncture. However, this approach is limited by poor visualization of the left atrial posterior border on digital subtraction angiography (DSA) and anatomical variability, which increases the risk of injury to the atrial posterior wall or aorta 15–21. To mitigate transseptal puncture complications, recent studies have reported the use of intracardiac echocardiography (ICE), transesophageal echocardiography (TEE), real-time magnetic resonance imaging (MRI), and rotational angiography for procedural guidance 22–25. Our technique integrates coronary sinus catheter guidance with spinal landmarks for dual-plane localization and demonstrates excellent performance.

The definition of the O point depends on the specific cardiac interventional procedure, including determining whether a superior, inferior, anterior, or posterior position is more favorable for the operation. For instance, during catheter ablation for atrial fibrillation, we prioritize the optimal distance from the right inferior pulmonary vein (RIPV). Therefore, in the craniocaudal direction, we select the center of the fossa ovalis, which is defined as the midpoint of the thinnest segment of the fossa ovalis in the coronal view. In the anteroposterior direction, we take the ostium of the right superior pulmonary vein (RSPV) and its course visualized on coronal images as references. Finally, using this method, we measure the distance between the O point and the spine on 3D-CT reconstructed images. Of note, when selecting the O point, we also need to consider the depth of the aortic impression and the degree of the spinal impression on the posterior wall of the left atrium, so as to minimize the risk of perforating the anterior or posterior atrial wall after transseptal puncture.

Compared with conventional transseptal puncture guided solely by two-dimensional DSA imaging, the left atrial CT three-dimensional reconstruction biplane positioning method offers several advantages. First, this dual-level approach utilizes horizontal and vertical spatial relationships for anatomical localization: the anteroposterior position is determined by the distance from the puncture point to the anterior edge of the spine, while the superior-inferior position is defined by the distance from the puncture point to the highest point of the great cardiac vein. Second, preoperative assessment of atrial anatomy can predict procedural difficulty; a greater distance between the aortic sinus and the left atrial posterior border facilitates puncture, and a larger superior-inferior left atrial diameter provides a wider safety margin. Third, this method is particularly advantageous in patients with left atrial structural abnormalities-such as a flattened anteroposterior diameter, reduced superior-inferior dimension, marked left atrial enlargement (diameter > 50 mm), or a distant inferior vena cava that prevents the needle from reaching the target-conditions that can be successfully managed using this approach. Finally, the technique allows flexible intraoperative selection of the puncture site, ensuring precision and facilitating subsequent interventions such as left atrial appendage occlusion, radiofrequency ablation, or cryoablation for atrial fibrillation.

This study found mean X/H and Y/H ratios of 0.8 ± 0.2 and 0.5 ± 0.1, respectively, indicating that the optimal puncture point is typically located 0.8 vertebral body widths anterior to the spine and 0.5 vertebral body heights above the highest point of the great cardiac vein. Furthermore, in the analysis stratified by left atrial diameter, Group 3 exhibited significantly higher X values than Groups 1 and 2. This may result from posterior displacement of the fossa ovalis due to left atrial enlargement, although anatomical validation is needed. These findings suggest that in patients with markedly enlarged left atria (>50 mm), the puncture site should be positioned farther from the spine.

During transseptal puncture, the posterior margin of the left atrium was not clearly visible under fluoroscopy in 17 patients, who had significantly higher BMIs than the remaining 83 patients. This indicates that obesity impairs visualization of the left atrial posterior border on x-ray. For such patients, the biplane CT-guided method offers superior safety and visualization compared to traditional fluoroscopy-only approaches. Additionally, preoperative left atrial CT three-dimensional reconstruction can detect structural features such as left atrial diverticula, atrial septal anomalies, left atrial roof veins, coronary sinus dimensions and course, esophageal position relative to the left atrial posterior wall, aortic sinus dilation, and coronary artery tortuosity adjacent to the pulmonary vein antra. It also enables preprocedural screening for left atrial appendage thrombus in patients with paroxysmal atrial fibrillation 26. Moreover, compared with ICE- or TEE-assisted techniques, our method enables centers lacking access to ICE or TEE to perform transseptal puncture safely, thereby reducing technical barriers and overall patient costs.

This feasibility study introduces a biplane positioning method guided by 3D-CT reconstruction of the left atrium, contributing to the advancement of image-guided structural heart interventions. By integrating preoperative CT-derived anatomical ratios with intraoperative fluoroscopy, the left atrial 3D-CT-guided biplane positioning method demonstrates feasibility, accuracy, and safety of TSP, including patients with enlarged atria, structural anomalies, or overweight. The protocol is feasible within a limited, single-center cohort.

Limitations of this technique: This study is single-center with a limited sample size and lacks a comparative control group. Preoperative CT imaging increases patient radiation exposure and contrast agent use. The left atrial CT three-dimensional reconstruction biplane method is particularly advantageous for patients with enlarged left atria and indistinct fossa ovalis “bounce” signs during puncture. For patients with normal-sized left atria, the traditional method is more convenient and avoids the cost of routine preoperative CT. Furthermore, this study did not include a direct comparison between the traditional and CT-guided biplane methods. Initial cases were selected for moderate procedural difficulty to ensure successful implementation, and future work will focus on accumulating experience with more complex anatomies. The CT-guided transseptal puncture site is susceptible to respiratory motion; therefore, imaging was acquired during end-expiration. Additionally, double-atrium CTA which could better delineate the anatomical position of the PFO was not performed, warranting further investigation.

## Data Availability

The raw data supporting the conclusions of this article will be made available by the authors, without undue reservation.
